# Observation of different reactivities of *para* and *ortho-*water towards trapped diazenylium ions

**DOI:** 10.1038/s41467-018-04483-3

**Published:** 2018-05-29

**Authors:** Ardita Kilaj, Hong Gao, Daniel Rösch, Uxia Rivero, Jochen Küpper, Stefan Willitsch

**Affiliations:** 1 Department of Chemistry, University of Basel, Klingelbergstrasse 80, Basel, 4056 Switzerland; 2 Center for Free-Electron Laser Science, Deutsches Elektronen-Synchrotron DESY, Notkestrasse 85, Hamburg, 22607 Germany; 3 Department of Physics, Universität Hamburg, Luruper Chaussee 149, Hamburg, 22761 Germany; 4 Department of Chemistry, Universität Hamburg, Martin-Luther-King-Platz 6, Hamburg, 20146 Germany; 5 The Hamburg Center for Ultrafast Imaging, Universität Hamburg, Luruper Chaussee 149, Hamburg, 22761 Germany; 6 Present Address: Beijing National Laboratory of Molecular Sciences, State Key Laboratory of Molecular Reaction Dynamics, Institute of Chemistry, Chinese Academy of Sciences, Beijing, 100190 China

## Abstract

Water is one of the most fundamental molecules in chemistry, biology and astrophysics. It exists as two distinct nuclear-spin isomers, *para-* and *ortho-*water, which do not interconvert in isolated molecules. The experimental challenges in preparing pure samples of the two isomers have thus far precluded a characterization of their individual chemical behavior. Capitalizing on recent advances in the electrostatic deflection of polar molecules, we separate the ground states of *para-* and *ortho-*water in a molecular beam to show that the two isomers exhibit different reactivities in a prototypical reaction with trapped diazenylium ions. Based on ab initio calculations and a modelling of the reaction kinetics using rotationally adiabatic capture theory, we rationalize this finding in terms of different rotational averaging of ion-dipole interactions during the reaction.

## Abstract (web summary)

Water molecules exist as two distinct nuclear-spin isomers denoted *ortho* and *para*. Here, the authors separate these two isomers in the gas phase to show that they exhibit different reactivities in a prototypical proton-transfer reaction.

## Introduction

Water, H_2_O, is one of the key molecules in nature, it acts as the fundamental solvent in biological systems and is one of the major molecular constituents of the universe. It exists in two forms, *para(p)-*water and *ortho(o)-*water, which are distinguished by their values of the quantum number of the total nuclear spin *I*, where *I* = 0 and 1 for *p-* and *o*-H_2_O, respectively. Interconversion of the nuclear-spin isomers is forbidden in isolated molecules and nuclear-spin symmetry is usually conserved in collisions, by electromagnetic radiation and even in chemical reactions^1–3^. However, nuclear-spin-symmetry interconversion has been observed in a variety of polyatomic molecules and has been rationalized to occur via doorway states with a mixed nuclear-spin character^2,4,5^. For water in the vapor and condensed phases, the *para/ortho-*interconversion rates reported in the literature vary widely and remain a controversial topic^6–8^.

Apart from their total nuclear spin, *para-* and *ortho-*water also differ in other important respects. Because the generalized Pauli principle dictates that the total molecular wavefunction has to be antisymmetric under the permutation of the two hydrogen nuclei in the molecule^1^, *(ortho-) para*-water is associated with (anti)symmetric rotational functions in the electronic-vibrational ground state of the molecule. Thus, nuclear-spin and rotational symmetry are intimately linked^9^. As a consequence, the ground state of *para-*water is the absolute rotational ground state |*j*_*K*__*a*__*K*__*c*_〉 = |0_00_〉, whereas the ground state of *ortho*-water is the first excited rotational state |*j*_*K*__*a*__*K*__*c*_〉 = |1_01_〉. Here, *j* denotes the quantum number of the rotational angular momentum and *K*_*a*_ and *K*_*c*_ are the quantum numbers of the projection of the rotational angular momentum on the *a* and *c* principal axes of inertia of the molecule, respectively.

Considering the different properties of its two nuclear-spin isomers and the eminent importance of water in a variety of chemical contexts, it begs the question whether *para-* and *ortho-*water also show different chemical behavior. In a wider context, this problem ties into ongoing efforts to understand how different molecular degrees of freedom (translation, nuclear spin, rotation, vibration, electronic motion) and the interplay between them influence chemical reactivity. Despite the significant amount of studies focusing on vibrational effects in chemical reactions^10–12^, the roles of nuclear spin and molecular rotation have scarcely been explored experimentally. This is mainly due to the fact that rotational energy transfer is likely to happen in any collision rendering it difficult to prepare molecules in specific rotational levels^13,14^ so that only comparatively few rotational-state resolved studies have been reported so far^15–17^. A similar scarcity of data exists with regard to studies involving individual nuclear-spin isomers^18^ which are in general difficult to separate and to prepare individually ^9,19^.

Recent progress in manipulating polar molecules using electrostatic fields has made it possible to select and spatially separate different conformers and rotational states of molecules in supersonic molecular beams^14^. By combining this technology with a stationary reaction target of Coulomb-crystallized ions in a linear-quadrupole ion trap (LQT)^20,21^, we have recently studied conformer-selected molecule-ion reaction dynamics and observed that reaction-rate constants can strongly depend on molecular conformation^22,23^. Here, we extend this method to the separation of different nuclear-spin isomers using their different rotational properties in order to study ion-molecule reactions with control over the rotational and nuclear-spin state of the neutral reaction partner, a field on which only scarce experimental data are available so far. Our approach is particularly suited for polyatomic neutrals for which rigorous rotational state preparation is difficult to achieve solely by supersonic cooling^16^. As an example, we investigate the proton-transfer reaction of water with ionic diazenylium (N_2_H^+^),

1 $${\mathrm{H}}_2{\mathrm{O}} + {\mathrm{N}}_2{\mathrm{H}}^ + \to {\mathrm{N}}_2 + {\mathrm{H}}_3{\mathrm{O}}^ + ,$$

an important molecule in astrochemistry which has been observed in the interstellar medium^24^. Its detection has proven crucial to trace molecular nitrogen in pre-stellar clouds to understand the early stages of star formation ^25,26^.

We find that the *para-*species reacts 23(9)% faster than the *ortho-*isomer. Based on ab initio calculations and a modelling of the reaction kinetics using rotationally adiabatic capture theory, we rationalize this finding in terms of different rotational averaging of ion-dipole interactions during the reaction.

## Results

### Experimental approach

The two ground states of *para-* and *ortho-*water show distinct responses to an electric field, i.e., different Stark-energy shifts and correspondingly different effective space-fixed dipole moments (Supplementary Note 1 and^9^). This enables their spatial separation by the electric field gradient of an electrostatic deflector ^9,14^.

**Fig. 1:**
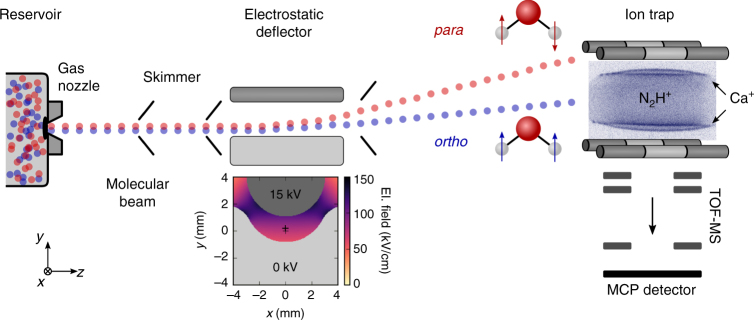
Schematic of the experimental setup. A pulsed molecular beam of water molecules seeded in argon emanates from a room-temperature reservoir through a pulsed gas nozzle and passes an electrostatic deflector. The inhomogeneous electric field inside the deflector (shown in the inset below) spatially separates *para-* and *ortho-*water molecules due to their different effective dipole moments. After the deflector, the beam is directed at an ion trap containing a Coulomb crystal of Ca^+^ and sympathetically cooled N_2_H^+^ reactant ions (inset image). The products and kinetics of reactive collisions between N_2_H^+^ and H_2_O are probed using a time-of-flight mass spectrometer (TOF-MS)^27^

The experimental setup is schematically depicted in Fig. 1. It consists of a molecular-beam machine equipped with the electrostatic deflector connected to an ultrahigh-vacuum chamber housing an ion trap^23,27^. A beam of internally cold molecules was formed in a pulsed supersonic expansion of water seeded in argon carrier gas (Methods). The molecular beam was collimated by two skimmers before entering the electrostatic deflector. A voltage of 15 kV was applied across the deflector electrodes in order to generate a vertical electric field gradient for the spatial separation of the two nuclear-spin isomers. After passing another skimmer, the beam was directed towards a LQT. The trap was loaded with Coulomb crystals of laser-cooled Ca^+^ ions^20,22^ as well as sympathetically cooled N_2_H^+^ reactant ions (image inset in Fig. 1). By vertically tilting the molecular beam apparatus relative to the LQT, different regions of the deflected molecular beam were overlapped with the Coulomb crystals^23^. The tilting angle of the molecular beam apparatus defined a deflection coordinate *y* for molecules arriving at the trap center. After exposure to the molecular beam for a variable time period, the Coulomb crystals were ejected into a high-resolution time-of-flight mass spectrometer (TOF-MS)^27^ for the mass and quantitative analysis of their constituents.

### Isomer populations in the deflected water beam

In order to probe its composition in terms of quantum states and to characterize the spatial separation of the two nuclear-spin isomers, density profiles of the molecular beam were measured. A pulsed ultraviolet laser beam was used to generate water ions by (2+1) resonance-enhanced multi-photon ionization (REMPI) via selected rotational levels of the ${\tilde{\mathrm C}}$ electronic state^28^. The ions were subsequently ejected into the TOF-MS. This technique enabled the selective detection of the ground states of either *para-* or *ortho-*water and the determination of the individual density profiles of the two isomers in the beam. From a REMPI spectrum of an undeflected water beam, it was confirmed that the supersonic expansion was composed predominantly of the *j*_*K*__*a*__*K*__*c*_ = 0_00_ and 1_01_ rotational states of H_2_O, i.e., the ground states of *p-* and *o-*H_2_O, respectively. A possible minor contribution from the 1_10_ state did not interfere with the present experiments (Supplementary Note 2 and 3).

**Fig. 2:**
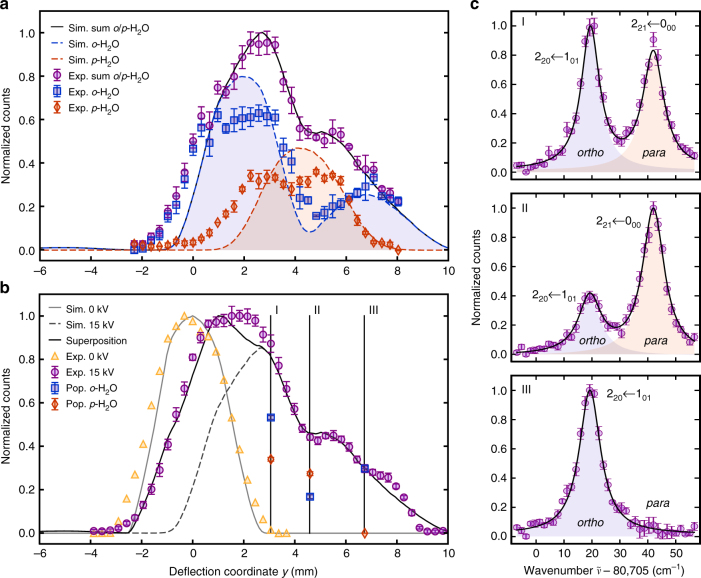
Molecular-beam deflection profiles of the ground states of *para* and *ortho-*water. **a** Experimental isomer-specific density profiles of *o-* (blue squares) and *p-*H_2_O (red diamonds) in the deflected molecular beam (deflector voltage 15 kV) measured by (2 + 1) REMPI together with the total deflection profile (sum of the *ortho-* and *para-*signals, purple circles). The lines represent Monte-Carlo trajectory simulations of the deflection profiles. The contributions from *ortho-* and *para-*water are indicated by the blue and red shaded areas, respectively. **b** Total water deflection profile measured by femtosecond-laser ionization for deflector voltages of 0 kV (yellow triangles) and 15 kV (purple circles). The three vertical lines marked I, II, and III indicate the deflection coordinates at which reaction rates were measured. The red/blue symbols represent the relative populations of the isomers normalized to the total signal at positions I, II, and III as determined from the REMPI spectra shown in **c**. **c** REMPI spectra of H_2_O measured at the three positions I, II, III (purple circles). The two peaks observed at 80,724 cm^−1^ and 80,747 cm^−1^ correspond to transitions from the ground states of *ortho-* and *para-*water, respectively. The peaks are fitted with a sum of two Lorentzians (solid black line) with contributions from *ortho-* and *para-*isomers depicted as blue and red shaded areas, respectively. Error bars correspond to one standard error of at least three independent measurements

Figure 2a shows deflection profiles of *para* (red diamonds) and *ortho-* (blue squares) water obtained from the ion signal at mass-to-charge ratio *m*/*z* = 18 u recorded as a function of the deflection coordinate *y* at a deflector voltage of 15 kV. The purple circles represent the sum of the *para* and *ortho-*profiles. The different projections of the angular momentum of the ground state of *ortho*-water onto the space-fixed direction of the electric field leads to two components, *M* = 0 and |*M*| = 1, which exhibit a weak and strong Stark shift, respectively. These correlate with the two peaks of the *o-*H_2_O deflection profile at low and high deflection coordinates, respectively. Contrarily, the *para-*isomer only has one angular-momentum projection component *M* = 0 with an intermediate Stark shift such that its deflection profile shows a single peak situated in between the two peaks of the *ortho-*form. In this way, a partial spatial separation of the two isomers was achieved and the *o*/*p*-ratio was well defined at each deflection coordinate^9^. The solid and broken lines show corresponding theoretical deflection profiles derived from Monte Carlo trajectory simulations (Supplementary Note 1).

In addition, a femtosecond (fs) laser was employed to probe molecules reaching the trap center by strong-field ionization irrespective of the species or the internal quantum state^29^. Subsequent ion ejection into the TOF-MS enabled the determination of the combined relative density of *para-* and *ortho-*water molecules in the beam as a function of the deflection coordinate. The acquired beam profiles for deflector voltages of 0 and 15 kV are presented in Fig. 2b as yellow triangles and purple circles, respectively. Note that in these measurements, the gas valve was operated at a higher repetition rate (200 Hz) compared to the experiments shown in Fig. 2a (20 Hz) which leads to slightly different molecular-beam characteristics and therefore deflection profiles. At 15 kV, experiment and simulation (dashed grey line) agree well at large deflection coordinates, but differ significantly around *y* = 0. In this region, the mass spectra indicate the presence of clusters formed in the supersonic expansion. A fs laser pulse can break these clusters resulting in water ions detected together with the water-monomer signal at *m*/*z* = 18 u in the TOF-MS. Our data also show that these clusters are not deflected and do not contaminate the beam at deflection coordinates larger than 2 mm (Supplementary Note 3). This picture is corroborated by the reproduction of the experimental beam profile by a weighted superposition (black solid line) of simulations of the deflected water beam at 15 kV and an undeflected beam at 0 kV (grey solid line).

Probing the specific reactivities of the two isomers requires the preparation of samples with well defined *para/ortho-*ratios. Based on the deflection profiles and their simulations, three deflection coordinates with varying relative populations of *para-* and *ortho-*water were chosen. At each of these positions labelled I, II, and III in Fig. 2b, REMPI spectra were recorded from which the populations of *para* and *ortho-*water were determined (Fig. 2c). From fits of the intensities of the lines in the spectra, the relative populations of the two isomers were obtained (Supplementary Note 2). The populations of *para* (*ortho-*) water thus obtained were 39(1)% (61(1)%), 62(2)% (38(2)%) and 0% (100%) at positions I, II, and III. We note that at position III, the beam consists of pure *ortho-*water within the measurement uncertainties and therefore enables a direct measurement of the reaction rate constant of *ortho-*water.

### Reaction-rate measurements

**Fig. 3:**
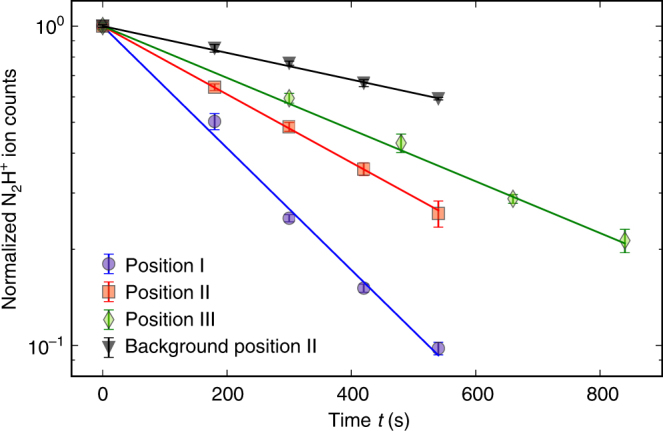
Reaction-rate measurements at the deflection coordinates I–III indicated in Fig. 2b. The data are normalized to the ion signal at time *t* = 0. The lines represent fits to the data according to an integrated pseudo-first-order rate law. The black triangles show an example of a measurement of the reaction rate with background gas at position II for comparison. Error bars correspond to one standard error of four independent measurements

With the molecular beam prepared with well-known ratios of the two isomers at different deflection coordinates, measurements of the rate of reaction (1) were performed (Methods). First, a Coulomb crystal of about 1000 Ca^+^ ions was loaded into the LQT. Then, nitrogen gas was leaked into the vacuum chamber and ${\mathrm{N}}_2^ +$ ions were generated by fs-laser ionization. After the formation of ${\mathrm{N}}_2^ +$ ions in the trap, H_2_ gas was leaked into the vacuum chamber to quantitatively convert ${\mathrm{N}}_2^ +$ into N_2_H^+^ via the reaction ${\mathrm{N}}_{\mathrm{2}}^ + + {\mathrm{H}}_{\mathrm{2}} \to {\mathrm{N}}_{\mathrm{2}}{\mathrm{H}}^ + + {\mathrm{H}}$. The thus formed N_2_H^+^ ions were sympathetically cooled into the Coulomb crystal and accumulated at its center (inset in Fig. 1). Subsequently, the molecular beam apparatus was set to a specific deflection coordinate and the deflector was turned on at a voltage of 15 kV to direct the molecular beam at the Coulomb crystal and engage the reaction. After a variable period of exposure to the molecular beam, the reduction of the number of N_2_H^+^ reactant ions was probed by ejecting the Coulomb crystal into the TOF-MS^27^ (Fig. 3). In addition, the formation of H_3_O^+^ as the ionic reaction product was verified using the TOF-MS. The rate measurements were repeated five times for each of the three deflection coordinates I–III. For every reaction measurement, a subsequent measurement of the rate of reaction of N_2_H^+^ with the background gas in the vacuum chamber was performed by setting the deflector voltage to 0 kV while keeping the deflection coordinate constant, thus pointing the molecular beam away from the center of the LQT. Since the number of water molecules is continuously replenished through the molecular beam, the rate constants could be determined within the framework of a pseudo-first-order kinetics treatment (Supplementary Note 4). The pseudo-first-order rate constants of the reactions with background gas were directly subtracted from the total rate constants to give the rate constants for the reactions of the diazenylium ions with water from the molecular beam.

### Isomer-specific rate constants

Combining the determinations of the total reaction-rate constants, of the relative populations of *o-* and *p-*H_2_O at positions I, II, and III, and of the combined density profile of the deflected beam, the individual reaction-rate constants *k*_*o*_ and *k*_*p*_ of *ortho-* and *para-*water, respectively, were deduced. For each of the three deflection coordinates *y*_*i*_ ($i \in \{ {\mathrm{I}},{\mathrm{II}},{\mathrm{III}}\}$), the total first-order rate constant $k^{\prime}_{{\mathrm{tot}},i}$ is given by

2 $$k^{\prime}_{{\mathrm{tot}},i} = \tilde n_i\left( {p_{o,i}\;k^{\prime}_o + p_{p,i}\;k^{\prime}_p} \right),$$

where *p*_*o*,*i*_ and *p*_*p*,*i*_ are the populations of *ortho-* and *para-*water, respectively, and $\tilde n_i$ are the relative densities of the water beam at positions *y*_*i*_. $\tilde n_i$ is obtained from the beam profile *I*(*y*) measured with the fs laser at 15 kV (Fig. 2b) via $\tilde n_i = I\left( {y_i} \right)/I\left( {y_{{\mathrm{II}}}} \right)$ with position II taken as reference point. Once $k^{\prime}_o$ and $k^{\prime}_p$ were known, the relative difference of the reaction rates of the two isomers was calculated as $r = 2\left( {k^{\prime}_p - k^{\prime}_o} \right)/\left( {k^{\prime}_p + k^{\prime}_o} \right)$.

The system of equations (2) was solved by a least-squares optimization. From the experimental data, the pseudo-first-order rate constants were determined to be $k^{\prime}_o = 1.4\left( 1 \right) \times 10^{ - 3}{\mathrm{s}}^{ - {\mathrm{1}}}$ and $k^{\prime}_p = 1.8\left( 1 \right) \times 10^{ - 3}{\mathrm{s}}^{ - {\mathrm{1}}}$ yielding a relative difference *r* = 23(9)% between the reactivities of the *para-* and *ortho-*isomer.

Absolute bimolecular reaction-rate constants *k*_*o*/*p*_ were calculated from $k_{o/p} = k^{\prime}_{o/p}/n_{{\mathrm{H}}_{\mathrm{2}}{\mathrm{O,II}}}$, where the total time-averaged beam density at the reference position II, $n_{{\mathrm{H}}_{\mathrm{2}}{\mathrm{O,II}}} = 3.0\left( 5 \right) \times 10^5{\mathrm{cm}}^{ - {\mathrm{3}}}$, was estimated according to the procedures described in Supplementary Note 5. Using this information, the absolute reaction rates obtained from this experiment are $k_o = 4.8\left( 9 \right) \times 10^{ - 9}{\mathrm{cm}}^{\mathrm{3}}{\mathrm{s}}^{ - {\mathrm{1}}}$ for *ortho-*water and $k_p = 6\left( 1 \right) \times 10^{ - 9}{\mathrm{cm}}^{\mathrm{3}}{\mathrm{s}}^{ - {\mathrm{1}}}$ for *para-*water, respectively.

## Discussion

**Fig. 4:**
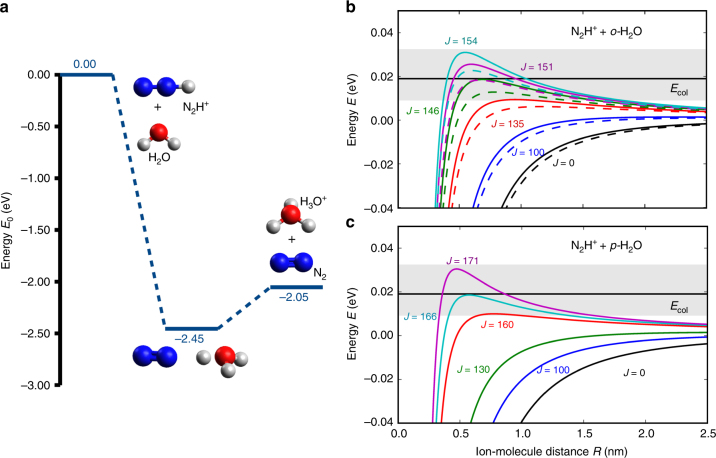
Theoretical predictions from ab initio calculations and adiabatic capture theory. **a** Potential-energy profile along the reaction coordinate for the proton transfer reaction between N_2_H^+^ and H_2_O at the CCSD/aug-cc-pVTZ level of theory. The relative energies with respect to the reactants as well as the structures of the stationary points are shown. Blue, red and white spheres represent nitrogen, oxygen, and hydrogen atoms. **b**, **c** Rotationally adiabatic, centrifugally corrected long-range interaction potentials for the reaction of the ground states of *o-* (**b**) and *p-* (**c**) H_2_O with N_2_H^+^ for different values of the total angular momentum quantum number *J*. In **b**, the dashed (solid) lines correspond to the |Ω| = 0(1) components of the *ortho-*ground state. The grey-shaded areas show an estimate of the uncertainty in the experimental collision energy *E*_col_ indicated by the black horizontal line

To understand the reason for the different reactivities of *para-* and *ortho-*water in the present case, ab initio calculations of the energy profile of the reaction were performed (Methods and Supplementary Note 6). As can be seen in Fig. 4a, the reaction was found to be barrierless and to proceed via the formation of an intermediate complex in which one hydrogen atom is shared between the nitrogen and water moieties. No transition state could be located within O–H^+^ distances ranging from 100 to 260 pm (see Supplementary Fig. 5). The energy of the products was found to be about 2 eV lower than the one of the reactants. This situation suggests that the kinetics can be modelled within the framework of a rotationally adiabatic quantum capture theory for barrierless ion-molecule reactions^30,31^. According to this approach, the reaction rates are entirely dominated by the properties of the long-range interaction potential of the reactants and centrifugal effects. All collisions up to a maximum total angular momentum *J*_max_ for which the relative kinetic energy exceeds the height of the centrifugal barrier lead to a successful reactive encounter (Supplementary Note 6).

For the present case, the relevant terms in the long-range interaction potential are the charge-induced dipole and charge-permanent dipole interactions. In the current experiments, both nuclear-spin isomers were cooled down to their relevant rotational ground states. The anisotropic nature of the ion-dipole interaction implies that it is sensitive to the rotational quantum state of the neutral molecule. Figure 4b, c shows rotationally adiabatic, centrifugally corrected interaction potentials for collisions of N_2_H^+^ with *o-* and *p-*H_2_O, respectively, as a function of the total angular momentum quantum number *J*. For the case of vanishing total angular momentum (*J* = 0), one can see that the potential-energy curves for the *ortho-*species are less steep than the one of the *para-*isomer, reflecting the stronger rotational averaging of the ion-dipole interaction in the ground state of *o*-H_2_O. For the $\left| {j_{K_aK_c}\left| \Omega \right|} \right\rangle = \left| {0_{00}0} \right\rangle$ ground state of the *para-*species, one obtains a maximum collisional angular-momentum *J*_max_ = 166 at the experimental collision energy *E*_col_ = 0.019 eV. Here, Ω is the quantum number of the projection of $\vec j$ on the distance vector between the ion and the neutral molecule in a body-fixed coordinate system describing the collision^30,31^. This value can be compared to *J*_max_ = 151 and *J*_max_ = 146 for the $\left| {1_{00}0} \right\rangle$ and $\left| {1_{01}1} \right\rangle$ states of the *ortho-*isomer, respectively.

The quantum capture model predicts a reaction-rate constant of $k_p^{{\mathrm{AC}}} = 5\left( 1 \right) \times 10^{ - 9}{\mathrm{cm}}^{\mathrm{3}}{\mathrm{s}}^{ - {\mathrm{1}}}$ for *para*-water compared to $k_o^{{\mathrm{AC}}} = 4.0\left( 9 \right) \times 10^{ - 9}{\mathrm{cm}}^{\mathrm{3}}{\mathrm{s}}^{ - {\mathrm{1}}}$ for *ortho-*water. The value for the *ortho-*species was obtained by summing over the contributions of all Ω components to the reaction cross section (Supplementary Note 6) assuming that the original preparation of *M* states of $o -$H_2_O in the deflector was scrambled during the transit of the molecules through the RF fields in the ion trap. In a classical interpretation of this result, the higher maximum angular momentum obtained for the *para-*isomer implies a larger impact parameter and, therefore, a higher reaction rate. The theoretical predictions agree with the measured values within their experimental uncertainties. The relative difference of the theoretical reaction-rate constants was calculated to be *r* = 24(5)% which can be compared with the experimental value of *r* = 23(9)%. The errors in the calculated rate constants arise from the uncertainty in the collision energy due to the experimental velocity distribution of the molecular beam and the micromotion of the ions in the large Coulomb crystals^20^ (Supplementary Note 7).

In conclusion, we have studied chemical reactions of the spatially separated ground states of *para-* and *ortho-*water with trapped diazenylium ions. We found a 23(9)% higher reactivity for the *para* nuclear-spin isomer which we attribute to the smaller degree of rotational averaging of the ion-dipole long-range interaction compared to the *ortho-*species. The observed difference in reactivities is thus a rotational effect which is induced by the nuclear-spin symmetry via the generalized Pauli principle. The present results highlight the interplay between nuclear-spin and rotational symmetry and its ramifications on chemical reactivity. They also provide an illustration of the effects of exchange symmetry on chemical processes which may be put in context with, e.g., its manifestations in the dynamics of ultracold collisions^32^. Measurements such as the ones presented here fill a gap in experimental investigations of ion-neutral reactions with control over the quantum states of the reactants. Indeed, to our knowledge the present study is the first in which rotationally state-selected polyatomic neutral molecules have been reacted with ions. The methods employed here are applicable to studies of a broad range of ion-neutral processes. They enable a quantitative understanding of how different molecular quantum states and, as demonstrated in our earlier study^22^, molecular conformations, influence chemical reactivity.

## Methods

### REMPI spectra

(2 + 1)-resonance-enhanced multiphoton-ionization (REMPI) spectra of H_2_O were acquired using the output of a frequency-doubled dye laser pumped by the 3rd harmonic (355 nm) of a Nd:YAG laser. The resulting laser pulses at 248 nm with an energy of about 1.5 mJ were focused into the molecular beam using a lens with a focal length of 30 cm. Details on the procedure for the analysis of the REMPI spectra are given in the Supplementary Note 1.

### Femtosecond-laser ionization and ion preparation

Strong-field ionization of H_2_O molecules was performed with pulses from a Ti:Sapphire femtosecond laser (CPA 2110, Clark-MXR, Inc.) at a wavelength of 775 nm and pulse duration of 150 fs focused down to a beam diameter of $\approx 50 \hskip2pt \mu {\mathrm{m}}$. Similarly, Ca atoms as well as N_2_ molecules (partial pressure 4.0 × 10^−9^ mbar) were ionized using the fs laser before loading into the ion trap in a standardized procedure generating crystals of a constant size and composition as verified by TOF-MS. The ${\mathrm{N}}_2^ +$ ions were reacted with H_2_ gas leaked into the ultrahigh-vacuum chamber for 30 s at a partial pressure of *p* = 2 × 10^−9^ mbar to yield N_2_H^+^ ions.

### Ion trapping and mass analysis

The ions were trapped in a radio frequency (RF) linear-quadrupole ion trap (LQT) operated at a peak-to-peak RF voltage *V*_RF,pp_ = 800 V and frequency Ω_RF_ = 2π × 3.304 MHz. Doppler laser cooling of Ca^+^ was achieved using two laser beams at 397 nm and 866 nm generated by frequency-stabilized external-cavity diode lasers^20^. Laser and sympathetic cooling as well as Coulomb crystallization of the ions were monitored by imaging the laser-cooling fluorescence of the Ca^+^ ions onto a camera coupled to a microscope (see sample image inset in Fig. 1). The LQT was connected to a TOF-MS orthogonal to the molecular-beam propagation axis for the mass and quantitative analysis of reactant and product ions ^27^.

### Molecular beam and Stark deflector

The molecular beam was generated from water vapor at room temperature and seeded in argon carrier gas at 3 bar. The gas mixture was pulsed through an Amsterdam cantilever piezo valve (ACPV2, 150 μm nozzle) at a repetition rate of 200 Hz and a pulse width of 30 μs. The velocity of the resulting molecular beam was measured to be 575(65) m/s. The electrostatic deflector consists of a pair of 15.4 cm long electrodes separated by 1.4 mm ^23^.

### Theory

Effective dipole moments and Stark energy curves of individual rotational states of water were calculated for input into Monte-Carlo trajectory simulations using the CMIstark software package^33^. Details of the numerical procedures are reported in the Supplementary Note 1. Ab initio calculations of the potential energy surface of the title reaction were performed using the Gaussian 09 suite of codes^34^. Geometry optimizations of stationary points were carried out at the CCSD/aug-cc-pVTZ level of theory.

### Code availability

The custom computer codes used for calculating Stark curves, for performing Monte Carlo trajectory simulations and for calculating adiabatic-capture rate constants are available from the corresponding author upon request.

### Data availability

The data that support the findings of this study are available from the corresponding author upon request.

## Electronic supplementary material

Supplementary Information
