# Supplementary material for: Observation of different reactivities of para and ortho-water towards trapped diazenylium ions
Source: Nat Commun. 2018 May 29;9:2096. doi: 10.1038/s41467-018-04483-3 (PMC5974139; doi:10.1038/s41467-018-04483-3)
Supplement: Supplementary file 1 — Supplementary Information [file 41467_2018_4483_MOESM1_ESM.pdf]

Supplementary Information: Observation of different reactivities of *para*- and *ortho*-water towards trapped diazenylium ions

Ardita Kilaj[1*], Hong Gao[1,2*], Daniel Rösch[1], Uxia Rivero[1], Jochen Küpper[3,4,5,6] and Stefan Willitsch[1†]

[1] Department of Chemistry, University of Basel, Klingelbergstrasse 80, 4056 Basel, Switzerland
[2] Present address: Beijing National Laboratory of Molecular Sciences, State Key Laboratory of Molecular Reaction Dynamics, Institute of Chemistry, Chinese Academy of Sciences, Beijing 100190, China
[3] Center for Free-Electron Laser Science, Deutsches Elektronen-Synchrotron DESY, Notkestrasse 85, 22607 Hamburg, Germany
[4] Department of Physics, Universität Hamburg, Luruper Chaussee 149, 22761 Hamburg, Germany
[5] Department of Chemistry, Universität Hamburg, Martin-Luther-King-Platz 6, 20146 Hamburg, Germany
[6] The Hamburg Center for Ultrafast Imaging, Universität Hamburg, Luruper Chaussee 149, * These authors contributed equally to the present work.
† Electronic mail: stefan.willitsch@unibas.ch

Supplementary Note 1. Analysis of REMPI spectra

In order to determine the rotational temperature of the undeflected water beam, a (2+1) REMPI spectrum was recorded by monitoring the ion signal detected at mass-to-charge ratio $m/z = 18$ u in the TOF-MS as a function of the UV laser wavelength (Supplementary Figure 1). The experimental spectrum was compared to a simulation using the software package PGOPHER [1]. Best agreement was found by assuming a rotational temperature of 7 K in line with previous studies [2, 3]. Two dominant transitions were identified, the $|j_{K_aK_c}\rangle = |2_{20}\rangle \leftarrow |1_{01}\rangle$ transition of *ortho*-water at 80724 cm^{-1} and the $|2_{21}\rangle \leftarrow |0_{00}\rangle$ transition of *para*-water at 80747 cm^{-1}. j, K_a, K_c denote the rotational quantum numbers of an asymmetric top. The experimental intensities of the lines, i.e., the areas A underneath the peaks, were determined from fits of a sum of Lorentz functions to the spectral features.

The relative line strengths S_o and S_p of the two transitions for *ortho*- and *para*-water, respectively, were estimated from the relevant peak areas A_o and A_p. Assuming that both nuclear-spin isomers were cooled down to their relevant ground states and that the *ortho:para* ratio of 3:1 prevalent in the water vapour prior to expansion was conserved in the molecular beam, we set

$$3 = \frac{A_\mathrm{o} S_\mathrm{p}}{A_\mathrm{p} S_\mathrm{o}}. \tag{S1}$$

With $S_\mathrm{o} = 1$, we obtained $S_\mathrm{p} = 1.51(1)$.

Finally, the populations p_σ ($\sigma \in \{o, p\}$) at a given deflection coordinate y_i ($i \in \{I, II, III\}$) were calculated from the peak areas $A_\sigma(y_i)$ and line strengths S_σ. From the scaled peak areas $\tilde{A}_\sigma(y_i) = A_\sigma(y_i)/S_\sigma$, we get

$$p_\sigma(y_i) = \frac{\tilde{A}_\sigma(y_i)}{\tilde{A}_\mathrm{p}(y_i) + \tilde{A}_\mathrm{o}(y_i)}. \tag{S2}$$

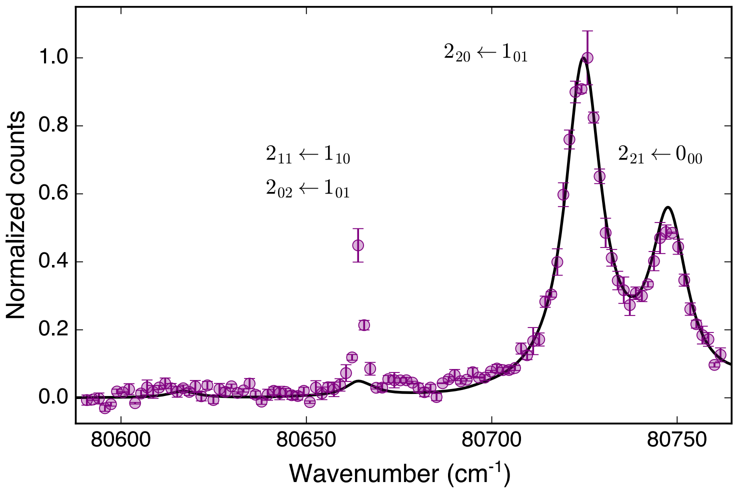

Supplementary Figure 1: **REMPI spectrum of water** (2+1) REMPI spectrum (data points) of a beam of water seeded in argon at a backing pressure of 3 bar and a simulation of the spectrum (line) at a rotational temperature of 7 K. Uncertainties correspond to one standard error.

The populations obtained using this method are listed in Supplementary Table 1.

Supplementary Note 2. Monte Carlo Trajectory Simulations

Effective dipole moments and Stark energies of the individual rotational states of water were calculated using the CMIstark software package [4]. Supplementary Figures 2a and c show simulated Stark energies and effective dipole moments, respectively, for the ground states of both isomers. All Stark components are high-field seeking with negative Stark shifts. The $|j_{K_aK_c}|M|\rangle = |1_{01}1\rangle$ state has the largest dipole moment resulting in the largest deflection in an electric field gradient, while $|1_{01}0\rangle$ shows the smallest Stark shift. Here, M denotes the quantum number of the space-fixed projection of $\vec{j}$. This results in a separation of the *ortho*-water $|M| = 0, 1$ states under electrostatic deflection. The value of the dipole moment of *para*-water in the $|0_{00}0\rangle$ state is in between those of *ortho*-water. Additionally, calculations of the Stark energies and dipole moments for the excited rotational states $|1_{10}\rangle$ and $|1_{11}\rangle$ are presented in Supplementary Figures 2b and d. Their contribution to the measured deflection profiles is discussed in Section Supplementary Note 3..

The state-specific deflection profiles of the molecular beam traveling through the entire setup including deflector (Figure 1 of the main text) have been simulated using a home-made software package based on CMIfly [5]. The trajectory simulations were carried out with 10^6 water molecules per quantum state. Their initial positions were uniformly sampled across the nozzle cross section. Their initial velocities were sampled from a normal distribution with a mean longitudinal velocity of 575 m/s and standard deviations of 10 m/s in the transverse directions and 69 m/s in the longitudinal direction. An *ortho:para* ratio of 3:1 was imposed on the molecular beam set by the nuclear-spin statistics. For each quantum state, simulated deflection profiles were obtained from histograms of the coordinates of the molecules upon arrival in the reaction region. Thermally averaged deflection profiles $n_{\sigma,T}(y)$ ($\sigma \in \{o, p\}$) for each spin isomer at rotational temperature T were calculated from the deflection profiles of the individual quantum states $n_{jK_aK_cM}(y)$ using the expression

$$n_{\sigma,T}(y) = \frac{g_\sigma}{N_\sigma} \sum_{j,K_a,K_c} \sum_{|M|=0}^{j} g_M g_{\sigma,K_aK_c} e^{-E_{jK_aK_c}/k_BT}\, n_{jK_aK_cM}(y), \tag{S3}$$

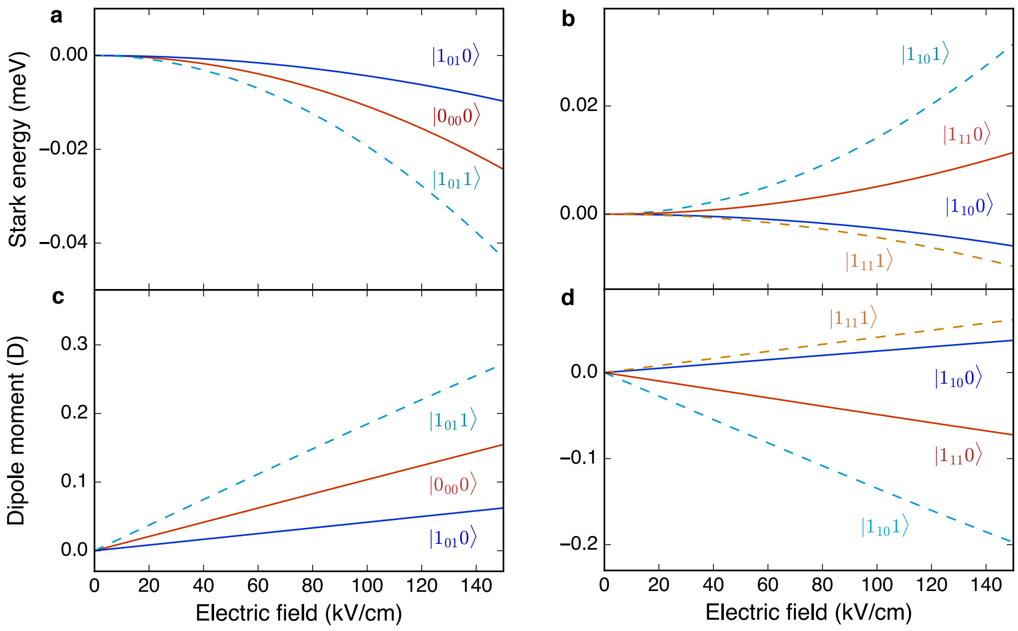

Supplementary Figure 2: **Stark shifts and effective dipole moments** Calculated stark energy shifts (a) and effective dipole moments (c) for the ground rotational states of *ortho*- ($|j_{K_aK_c}|M|\rangle = |1_{01}0\rangle, |1_{01}1\rangle$) and *para*- ($|0_{00}0\rangle$) water as a function of the electric-field strength. (b) and (d) Stark shifts and effective dipole moments for the higher excited rotational states $|1_{10}0\rangle, |1_{10}1\rangle$ and $|1_{11}0\rangle, |1_{11}1\rangle$.

with the partition function

$$N_\sigma = \sum_{j,K_a,K_c} \sum_{|M|=0}^{j} g_M g_{\sigma,K_aK_c} e^{-E_{jK_aK_c}/k_BT}. \tag{S4}$$

Here, k_B denotes the Boltzmann constant and $E_{jK_aK_c}$ are the field-free rotational energies. The degeneracy factor g_M takes values $g_M = 1$ for $M = 0$ and $g_M = 2$ for $|M| > 0$. The nuclear-spin symmetrization factors g_{σ,K_aK_c} are defined by

$$g_{\mathrm{o},K_aK_c} = \begin{cases} 1 & K_a + K_c \text{ odd} \\ 0 & K_a + K_c \text{ even} \end{cases} \tag{S5}$$

for the *ortho*- and

$$g_{\mathrm{p},K_aK_c} = \begin{cases} 0 & K_a + K_c \text{ odd} \\ 1 & K_a + K_c \text{ even} \end{cases} \tag{S6}$$

for the *para*-isomer. The nuclear-spin degeneracy factors are $g_\mathrm{p} = 1/4$ and $g_\mathrm{o} = 3/4$. The total thermal deflection profile was calculated from the sum of the deflection profiles of the *ortho*- and *para*-isomers,

$$n_{\mathrm{tot,T}}(y) = n_{\mathrm{para},T}(y) + n_{\mathrm{ortho},T}(y). \tag{S7}$$

Supplementary Note 3. Composition of the molecular beam

Contribution of water clusters: The mass spectra of the molecular beam recorded after fs-laser ionization indicate the presence of water clusters in the supersonic expansion. The fs laser is able to

ionize these clusters through strong-field ionization and break them into fragments including H_2O^+. The signal of these H_2O^+ cluster-fragment ions contributes to the $m/q = 18$ u signal of the water monomer ions in the mass spectra. This additional signal needs to be taken into account when evaluating the measured deflection profiles of water. Supplementary Figure 3 shows that cluster fragments with mass $m/z > 18$ u experience no significant deflection at a deflector voltage of 15 kV. We therefore assume that the detectable water clusters present in the molecular beam are largely undeflected and that the signal recorded at large deflection coordinates for $m/z = 18$ u is solely due to the water monomer. Thus, to avoid interference from reactions of the N_2H^+ ions with the clusters, the measurement positions I, II and III specified in the main text were chosen outside the region of deflection coordinates where undeflected water clusters would contribute to the rate measurements.

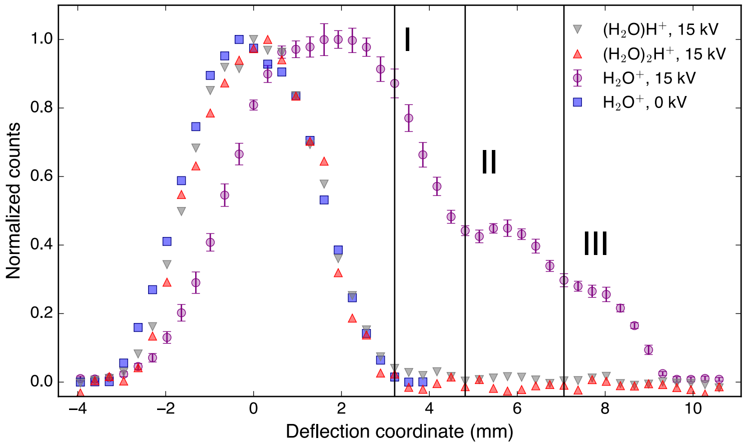

Supplementary Figure 3: **Deflection profiles of water clusters** Experimental deflection profiles of the water beam at a deflector voltage of 0 kV (blue squares) and 15 kV (purple circles) measured by fs-laser ionization. Deflection profiles of water clusters as detected by their fragment ions are also shown (grey and red triangles). These were produced from their parent molecules by fs-laser ionization and dissociation. No significant deflection is observed for these clusters. The vertical lines I, II and III specify the positions at which rate measurements were performed. Error bars correspond to one standard error.

Higher excited rotational states of water: The REMPI spectrum in Supplementary Figure 1 shows a weaker transition at 80664 cm^{-1} which could indicate the population of the higher excited rotational state $|1_{10}\rangle$ in the molecular beam. To assess a possible contribution of higher rotational states to the deflection profiles, we show in Supplementary Figure 4 simulations of a deflected beam at a rotational temperature of 30 K at which the $|1_{10}\rangle$ and $|1_{11}\rangle$ states are significantly populated. The Stark shifts and effective dipole moments of these states are shown in Supplementary Figures 2 b and d, respectively. In particular, these states exhibit low-field seeking Stark components which manifest themselves as shoulders in the deflection profile at negative deflection coordinates. As can be seen in Supplementary Figures 4, the experimental deflection profile exhibits no such features. We therefore conclude that higher excited rotational states do not play a significant role in the present experiments and that the measured reaction-rate constants predominantly reflect the contributions from the ground states of *para-* and *ortho*-water.

Supplementary Note 4. Reaction-rate constants

Determination of pseudo-first order rate constants: Pseudo-first-order reaction-rate constants were measured at the deflection coordinates I, II and III indicated in Figure 2b in the main text. At

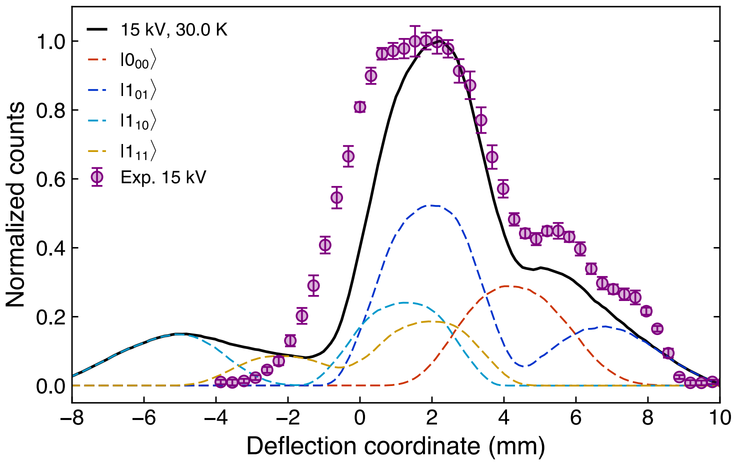

Supplementary Figure 4: **Simulated deflection profiles at higher temperature** Comparison of the deflection profile at 15 kV measured by fs laser ionization with a trajectory simulation at rotational temperature of 30 K. At this temperature, the partly low-field seeking states 1_{10} and 1_{11} are significantly populated. The simulation predicts a shoulder appearing at negative deflection coordinates. This is not observed in the experiment indicating that these higher rotational states are not significantly populated. Error bars correspond to one standard error.

each of these positions, five independent sets of data were taken each consisting of one measurement with a deflected molecular beam (deflector voltage 15 kV) and one background measurement (deflector voltage set to 0 kV). For each data set, the N_2H^+ ion signal $N(t)$ was recorded as a function of reaction time t in three consecutive measurements. The data were analyzed according to an integrated pseudo-first-order rate law

$$\ln\left(\frac{N(t)}{N(0)}\right) = -k't \tag{S8}$$

with the rate constant k' determined from a least-squares fit. For every data set, the pseudo-first-order rate constant of the reaction was calculated from the difference of the rate constants obtained with the molecular beam and the background measurement.

Determination of the rate constants of o/p-H_2O: From the measurements of the *ortho*/*para*-populations in the molecular beam using (2+1) REMPI and the pseudo-first-order rate constants at the three positions I, II and III, the pseudo-first-order rate constants k'_o for *ortho*- and k'_p for *para*-water were determined from the set of linear equations ($i \in \{\mathrm{I}, \mathrm{II}, \mathrm{III}\}$)

$$k'_i = \tilde{n}_i(p_{o,i}\, k'_o + p_{p,i}\, k'_p). \tag{S9}$$

The relative beam densities are defined as $\tilde{n}_i = I_i/I_{\mathrm{II}}$ where I_i is the normalized ion signal of the deflected molecular beam at deflection coordinate y_i obtained by fs-laser ionization. The parameters defining the equations are summarized in Supplementary Table 1.

The pseudo-first-order rate constants for *ortho*- and *para*-water, k'_o and k'_p, respectively, were obtained from a least-squares fit to the over-determined set of linear equations (S9) by minimising the cost function

$$S(k_o, k_p) = \sum_i (k'_i - \tilde{n}_i(p_{o,i}\, k_o + (1 - p_{o,i})\, k_p))^2. \tag{S10}$$

The resulting pseudo-first-order reaction-rate constants are given in the first column of Supplementary Table 2. The relative difference of the *para*- and *ortho*-rate constants was calculated using the

Region i	Pseudo 1st oder rates k'_i [s^{-1}]	Populations $p_{o,i} : p_{p,i}$	Normalized ion signal I_i
I	$3.13(5) \cdot 10^{-3}$	$0.61(1) : 0.39(1)$	0.87(4)
II	$1.63(4) \cdot 10^{-3}$	$0.38(2) : 0.62(2)$	0.44(1)
III	$0.95(5) \cdot 10^{-3}$	$1 : 0$	0.30(2)

Supplementary Table 1: **Parameters for data analysis** Total pseudo-first-order reaction-rate constants k'_i, nuclear-spin isomer populations p_σ and normalized ion signal I_i at the three positions $i =$ I, II and III.

expression

$$r = 2\frac{k_p - k_o}{k_p + k_o}. \tag{S11}$$

	k'[s^{-1}]	k[cm^3s^{-1}]	k_{AC} [cm^3s^{-1}]
o-H_2O	$1.4(1) \cdot 10^{-3}$	$4.8(9) \cdot 10^{-9}$	$4.0(6) \cdot 10^{-9}$
p-H_2O	$1.8(1) \cdot 10^{-3}$	$6(1) \cdot 10^{-9}$	$5(1) \cdot 10^{-9}$
Rel. difference r	23(9)%		24(5)%

Supplementary Table 2: **Rate constants** Experimental pseudo-first-order and second-order rate constants k' and k, respectively, and theoretical adiabatic capture-rate constants k_{AC} for the reaction of N_2H^+ with o- and p-H_2O.

Supplementary Note 5. Density of the molecular beam

The time-averaged density of water molecules in the molecular beam, $n_{MB,avg}$, relates to the density of molecules in one gas pulse, $n_{MB,pulse}$, via

$$n_{MB,avg} = n_{MB,pulse} f_{rep} \tau_{pulse}, \tag{S12}$$

where $f_{rep} = 200$ Hz is the repetition rate of the gas valve and $\tau_{pulse} = 487\mu$s is the FWHM of the temporal width of the molecular beam pulse in the reaction region.

To estimate $n_{MB,pulse}$, the fs-laser ionization signal I_{II} of H_2O from the molecular beam obtained at deflection position II was compared with the signal I_{BG} obtained from ionization of water in the background gas of the UHV chamber. Assuming that the ionization volumes V_{MB} and V_{BG} were equal (the diameter of the molecular beam of 4 mm was larger than the laser-focus diameter of ≈ 50 μm), $n_{MB,pulse}$ was estimated according to

$$n_{MB,pulse} = n_{BG}\frac{I_{II}}{I_{BG}}\underbrace{\frac{V_{MB}}{V_{BG}}}_{\approx 1} = n_{BG}\frac{I_{II}}{I_{BG}} \tag{S13}$$

Here, the density of water molecules in the background gas, n_{BG}, was estimated from a comparison of the measured pseudo-first-order rate constant of the reaction of background water with N_2H^+ ions with a literature value for the second-order rate constant at 300 K:

$$n_{BG} = k'_{BG}/k_{lit,300K}, \tag{S14}$$

with $k_{lit,300K} = 2.6(4) \times 10^{-9}$ cm^3s^{-1} taken from [6]. The value of the time-averaged beam density thus obtained is $n_{MB,avg} = 3.0(5) \times 10^5$ cm^{-3}. This value is in line with previous results [3] after adjustment for the different repetition rates of the experiments.

Supplementary Note 6. Collision velocity

In the present experiments, the collision energy was dominated by two contributions: the velocity of the H_2O molecules in the molecular beam and the velocities of the N_2H^+ ions in the Coulomb crystals.

Velocity of the molecular beam: The velocity of the molecular beam was determined by probing its time-resolved density profile in the ion-trap region at different longitudinal positions of the gas valve. The velocity spread was determined from the half-width-at-half-maximum (HWHM) of the temporal profile of the gas pulse which was assumed to be limited by the longitudinal velocity distribution of the molecules. The velocity of the molecular beam was determined to be v_{beam} =575(50) m/s with a HWHM of δv_{beam} = 65(36) m/s. This beam velocity was used for the adiabatic-capture-rate calculations and the Monte Carlo trajectory simulations.

Ion velocities: The velocities of Coulomb-crystallized ions in RF traps are usually dominated by their micromotion, i.e., their fast motion driven by the RF fields [7]. The contribution of their secular motion can be neglected because of laser and sympathetic cooling. The spread of micromotion velocities across the crystal thus defines the velocity spread δv of the entire ion ensemble. The micromotion velocities scale with the radial position of the ions in the trap [8]. They are zero for ions located in the trap centre and maximal for the outermost ions of the crystal. The radial extension r_0 of N_2H^+ ions can directly be determined from the edges of the non-fluorescing central region of the crystal images taken during the measurements (see inset in Figure 1 of the main text). Using the analytical expressions of the ion trajectories in a quadrupole trap [8], the root-mean-square velocity for the outermost ions in the present N_2H^+ crystals at $r_0 = 200$ μm was estimated to be $v_{\mathrm{rms}} = 163$ m/s. The corresponding micromotion energy was found to be $E_{\mathrm{kin}} = k_B \cdot 47$ K. Thus, the total uncertainty in the experimental collision velocity was calculated to be

$$\delta v = \sqrt{v_{rms}^2 + \delta v_{beam}^2} = 175 \text{ m/s}. \tag{S15}$$

Supplementary Note 7. Theory

Adiabatic capture theory: Rotationally adiabatic-capture rate constants were calculated using the theory developed by Clary and co-workers [9, 10]. A collision velocity $v = 575(175)$ m/s was assumed in the calculations. The long-range ion-molecule interaction potential adapted in the calculations consisted of the charge-induced dipole and charge-permanent dipole terms:

$$V(R,\beta) = -\frac{\alpha q^2}{2R^4} - \frac{q\mu_D \cos\beta}{R^2}, \tag{S16}$$

with q the ion charge, α the molecular isotropic polarisability, μ_D the permanent dipole moment of the neutral molecule and μ the reduced mass of the collision partners. The ion-molecule distance is denoted R and the orientation angle of the molecular dipole relative to the ion-molecule axis is denoted β. The molecular parameters for water used in this calculation are given in Supplementary Table 3.

Following [10], a set of rotationally adiabatic, centrifugally corrected potential energy curves $V_{JjK_aK_c\Omega}(R)$ was calculated (Figure 4b and c of the main text), where J denotes the quantum number of total angular momentum of the system, Ω the quantum number of its projection on the intermolecular axis and R the intermolecular distance. It is assumed that all collisions up to a maximum angular momentum J_{max} for which the collision energy E_{col} exceeds the centrifugal barrier of the effective potential lead to a successful reactive encounter. Hence, the reaction cross section is calculated to be [10]

$$\sigma(j, K_a, K_c, E_c) = \frac{\pi\hbar^2}{2\mu E_c}\,\frac{1}{2j+1}\sum_{\Omega=-j}^{j}(J_{\max}(j, K_a, K_c, \Omega)+1)^2. \tag{S17}$$

Rotational constants	A	27.88071 cm^{-1}
	B	14.52181 cm^{-1}
	C	9.27773 cm^{-1}
Reduced mass	μ	11.11533 u
Dipole moment	μ_D	1.85 D
Polarisability	α	1.45Å^3

Supplementary Table 3: **Molecular parameters** Molecular constants of water [11] used in the adiabatic-capture calculations.

The state-selective reaction-rate constants are then given by $k(j, K_a, K_c, E_c) = \sigma(j, K_a, K_c, E_c)v$. The calculated adiabatic-capture rate constants k_{AC} for the ground states of the two nuclear-spin isomers of water and their relative difference r are shown in Supplementary Table 2 together with the experimental values. The quoted uncertainties of the theoretical rate constants reflect the spread δv of the collision velocity v (Section Supplementary Note 6.) and were calculated from $\delta k = (k(v + \delta v) - k(v - \delta v))/2$.

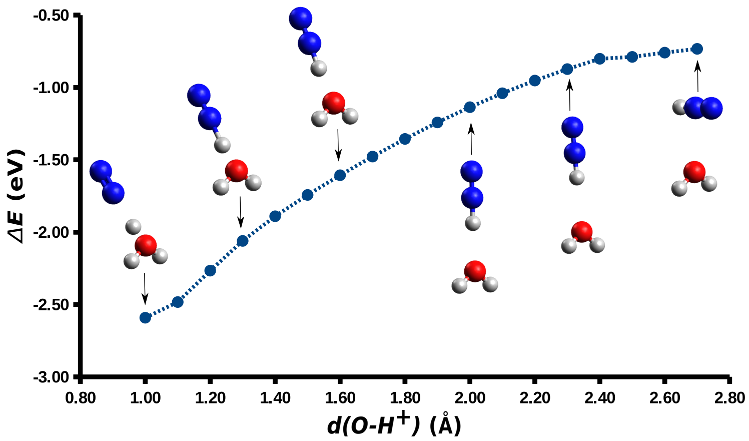

Supplementary Figure 5: **Reaction path** Optimized structures along the reaction coordinate of the proton transfer between N_2H^+ and H_2O at the CCSD/aug-cc-pVTZ level of theory. The energy is relative to that of the reactants. Blue spheres represent nitrogen atoms, red spheres oxygen atoms and white spheres hydrogen atoms.

Quantum-chemical calculations: In order to test whether the reaction $H_2O + N_2H^+ \rightarrow H_3O^+ + N_2$ is indeed barrierless, electronic structure calculations were carried out. We assumed for this purpose that the O-H$^+$ distance approximates the reaction coordinate. We constrained this distance while optimizing all other internal coordinates at the CCSD/aug-cc-pVTZ level of theory. The resulting electronic energies for the optimized structures along the O-H$^+$ coordinate are shown in Supplementary Figure 5. Over the full range of O-H$^+$ distances investigated, the energy continuously decreases from the reactants towards the products (from right to left in Supplementary Figure 5). These results indicate that the reaction is indeed barrierless and vindicate the application of capture theory to model the reaction kinetics.

Supplementary References

[1] Western, C. M. A program for simulating rotational, vibrational and electronic spectra. *J. Quant. Spectrosc. Radiat. Transf.* **186**, 221 (2016).

[2] Yang, C.-H., Sarma, G., ter Meulen, J. J., Parker, D. H. & Western, C. M. REMPI spectroscopy and predissociation of the $\tilde{C}^1B_1(v=0)$ rotational levels of H_2O, HOD and D_2O. *Phys. Chem. Chem. Phys.* **12**, 13983 (2010).

[3] Horke, D. A., Chang, Y.-P., Dlugolecki, K. & Küpper, J. Separating para and ortho water. *Angew. Chem. Int. Ed.* **53**, 11965 (2014).

[4] Chang, Y.-P., Filsinger, F., Sartakov, B. G. & Küpper, J. Cmistark: Python package for the stark-effect calculation and symmetry classification of linear, symmetric and asymmetric top wavefunctions in dc electric fields. *Comp. Phys. Comm.* **185**, 339 (2014).

[5] Chang, Y.-P., Horke, D. A., Trippel, S. & Küpper, J. Spatially-controlled complex molecules and their applications. *Int. Rev. Phys. Chem.* **34**, 557 (2015).

[6] Betowski, D., Pazyant, J. D., Mackay, G. I. & Bohme, D. K. Rate coefficient at 297 K for proton transfer reactions with H_2O. Comparisons with classical theories and exothermicity. *Chem. Phys. Lett.* **31**, 321 (1975).

[7] Willitsch, S. Coulomb-crystallised molecular ions in traps: methods, applications, prospects. *Int. Rev. Phys. Chem.* **31**, 175–199 (2012).

[8] Major, F. G., Gheorghe, V. N. & Werth, G. *Charged Particle Traps* (Springer, Berlin and Heidelberg, 2005).

[9] Clary, D. Rate constants for the reactions of ions with dipolar polyatomic molecules. *J. Chem. Soc., Faraday Trans. 2* **83**, 139–148 (1987).

[10] Stoecklin, T., Clary, D. C. & Palma, A. Rate constant calculations for ion-symmetric top and ion-asymmetric top reactions. *J. Chem. Soc. Faraday Trans.* **88**, 901 (1992).

[11] DeLucia, F., Helminger, P. & Kirchhoff, W. Microwave spectra of molecules of astrophysical interest V. water vapor. *J. Phys. Chem. Ref. Data* **3**, 211 (1974).
